# Puerarin Inhibits Proliferation, Migration and Invasion of Colon Cancer Cells and Induces Apoptosis via Suppression of the PI3K/AKT Signaling Pathway

**DOI:** 10.3390/ph18091378

**Published:** 2025-09-16

**Authors:** Lin Chen, Xuhong Li, Shijie Zhao, Mengyu Hao, Heng Wang, Zhi Zhou, Xinyu Xiong, Die Yuan, Piao Luo, Luwen Wang, Di Pan, Xiangchun Shen, Yue Zhang, Yan Chen

**Affiliations:** 1 State Key Laboratory of Discovery and Utilization of Functional Components in Traditional Chinese Medicine, School of Pharmaceutical Sciences, Guizhou Medical University, No. 6 Ankang Avenue, Guian New District, Guiyang 561113, China; chenlinzy@outlook.com (L.C.); lixuhong2023@126.com (X.L.); zhaoshijieyx@126.com (S.Z.); wlw757959@163.com (L.W.); pandipharm@126.com (D.P.); shenxiangchun@126.com (X.S.); 2 Key Laboratory of Natural Product Composition and Efficacy of Qiannan Prefecture, Qiannan Medical College for Nationalities, Duyun 558000, China; haomengyu163@163.com (M.H.); .; z1280303877@163.com (Z.Z.); daitou12138@163.com (X.X.); yuandieyx@163.com (D.Y.); luopiaoxp@126.com (P.L.); 3 The High Efficacy Application of Natural Medicinal Resources Engineering Center of Guizhou Province, School of Pharmaceutical Sciences, Guizhou Medical University, No. 6 Ankang Avenue, Guian New District, Guiyang 561113, China; 4 The Key Laboratory of Optimal Utilization of Natural Medicine Resources, School of Pharmaceutical Sciences, Guizhou Medical University, No. 6 Ankang Avenue, Guian New District, Guiyang 561113, China; 5 Key Laboratory of Novel Anti-Cancer Drug Targets Discovery and Application, School of Pharmaceutical Sciences, Guizhou Medical University, No. 6 Ankang Avenue, Guian New District, Guiyang 561113, China

**Keywords:** puerarin, colon cancer, PI3K/AKT, epithelial–mesenchymal transition, apoptosis

## Abstract

**Background**: Colon cancer is one of the most prevalent gastrointestinal malignancies worldwide, with high mortality and limited therapeutic options. Puerarin, a flavonoid compound derived from *Pueraria lobata*, has shown anticancer potential, but its molecular mechanisms against colon cancer remain unclear. **Methods and Results**: In this study, human colon cancer Caco-2 cells were treated with various concentrations of puerarin. Cell proliferation, migration, invasion, epithelial–mesenchymal transition (EMT), and apoptosis were evaluated using CCK-8, wound healing, Transwell, immunofluorescence, flow cytometry, and Western blot assays. Puerarin significantly inhibited Caco-2 cell proliferation in a dose- and time-dependent manner. It suppressed migration and invasion by increasing E-cadherin and reducing Vimentin expression. Apoptosis was induced via upregulation of BAX and downregulation of Bcl-2. Network pharmacology and KEGG analysis suggested PI3K/AKT signaling as a core regulatory pathway. Western blotting confirmed that puerarin reduced phosphorylation of PI3K and AKT. PI3K activator 740 Y-P promoted EMT and inhibited apoptosis, whereas puerarin and the PI3K inhibitor LY294002 reversed these effects. **Conclusions**: Puerarin exerts significant antitumor effects on Caco-2 colon cancer cells by inhibiting proliferation, migration, and EMT, while promoting apoptosis. These effects are mediated primarily through suppression of the PI3K/AKT signaling pathway. This study provides a theoretical basis for the use of puerarin as a natural therapeutic agent in colon cancer treatment.

## 1. Introduction

Colon cancer remains one of the leading and deadliest cancers worldwide [1]. In 2023, approximately 153,020 new cases of colon cancer were reported globally, resulting in 52,550 deaths. Of these, 19,550 diagnoses and 3750 deaths occurred in individuals under the age of 50 [2]. Alarmingly, global colon cancer rates are projected to more than double by 2035, particularly in low- and middle-income countries, where the aging population is expected to contribute significantly to the rising incidence [3]. In countries like China, the increasing prevalence of colon cancer has become a major public health concern.

At present, the standard treatment for early-stage colon cancer is surgical resection. However, as the disease is often diagnosed at more advanced stages, chemotherapy remains the primary treatment option for most patients. Common chemotherapy drugs, such as 5-fluorouracil (5-FU), oxaliplatin, and irinotecan, are frequently used in combination. Despite their therapeutic benefits, these agents have limited efficacy due to their toxicity to normal cells and the development of resistance in cancer cells [4]. As a result, there is an urgent need for the discovery of more effective and less toxic treatments, particularly those derived from traditional Chinese medicine.

Natural products, particularly those derived from plants, have long been valued for their accessibility, low toxicity, and therapeutic potential in treating a variety of diseases. Among these, flavonoids have garnered considerable attention due to their wide array of biological activities, such as anticancer, anti-inflammatory, and antioxidant properties [5,6,7]. Puerarin, a flavonoid extracted from *Pueraria lobata*, has demonstrated significant promise in the treatment of cardiovascular diseases since its clinical approval in 1993 [8]. More recently, there has been growing interest in puerarin’s anticancer potential, showing remarkable anti-tumor effects in animal models and a range of cancer cell lines [9,10]. However, the precise mechanisms by which puerarin influences colon cancer Caco-2 cells, particularly its effects on migration, invasion, EMT, and apoptosis, remain insufficiently explored, highlighting the need for further research.

The PI3K/AKT signaling pathway is a critical regulator of cancer progression, influencing key processes such as cell proliferation, invasion, metastasis, epithelial–mesenchymal transition (EMT), and apoptosis resistance [11,12,13]. Aberrant activation of this pathway contributes to tumorigenesis, making it a promising therapeutic target for cancer treatment [14]. Foundational overviews of PI3K/AKT biology and druggability in oncology further support the rationale to examine whether a natural compound like puerarin may exert its actions, at least in part, via suppression of this pathway [15,16,17]. This study evaluates how puerarin modulates cell migration, invasion, EMT, and apoptosis in Caco-2 colon cancer cells through the PI3K/AKT pathway. The results are expected to offer new insights into the anti-tumor mechanisms of puerarin and support the development of novel clinical strategies for treating colon cancer.

## 2. Results

### 2.1. Effects of Puerarin on the Viability and Proliferation of Colon Cancer Caco-2 Cells

**Figure 1:**
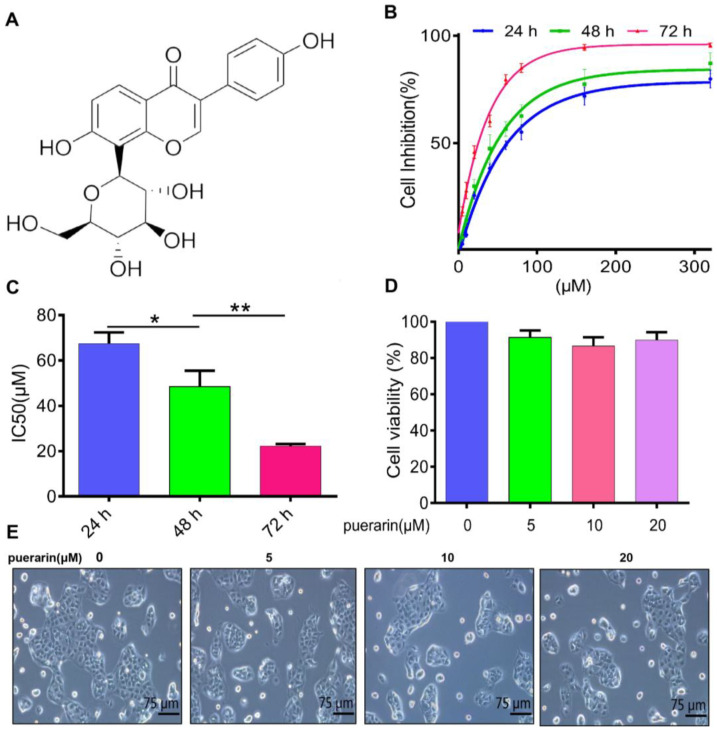
Effects of Puerarin on the viability and proliferation of Caco-2 cells. (**A**) Chemical structure of Puerarin (7,4′-dihydroxy-8-β-D-glucosylisoflavone; molecular formula C_21_H_20_O_9_). (**B**) CCK-8 assay showing Caco-2 cell viability after treatment with Puerarin (0–320 μM) for 24, 48, and 72 h (*n* = 5). (**C**) IC_50_ values of Puerarin at 24, 48, and 72 h (*n* = 5). (**D**) Puerarin at 5, 10, and 20 μM showed no significant effect on Caco-2 cell viability after 48 h (*n* = 5). (**E**) Morphology of Caco-2 cells remained unchanged with Puerarin at 5, 10, and 20 μM after 48 h; 100×; scale bar, 75 μm (*n* = 5). Statistical significance: * *p* < 0.05, ** *p* < 0.01.

Puerarin (Figure 1A, C_21_H_20_O_9_, 7,4′-dihydroxy-8-β-D-glucosylisoflavone), a flavonoid extracted from the traditional Chinese herb *Pueraria lobata*, significantly decreased the viability of human colon cancer Caco-2 cells. The CCK-8 assay was utilized to assess cell viability after exposure to a range of puerarin concentrations (0, 5, 10, 20, 40, 60, 80, 160, and 320 μM) for 24, 48, and 72 h. The data demonstrated a dose- and time-dependent decline in cell viability (Figure 1B). The IC_50_ values at 24, 48, and 72 h were 67.48 ± 4.98 μM, 48.47 ± 7.04 μM, and 22.21 ± 0.93 μM, respectively, showing statistically significant differences across the time points (Figure 1C). Notably, no morphological changes or effects on cell survival were observed at puerarin concentrations of 5, 10, and 20 μM after 48 h of treatment, indicating minimal cytotoxicity at lower doses (Figure 1D). Therefore, 5, 10, and 20 μM concentrations were chosen for subsequent experiments. These findings suggest that puerarin exerts potent inhibitory effects on Caco-2 cells, with lower concentrations showing minimal toxicity.

### 2.2. Effects of Puerarin on Migration, Invasion, and Epithelial–Mesenchymal Transition (EMT) in Caco-2 Cells

**Figure 2:**
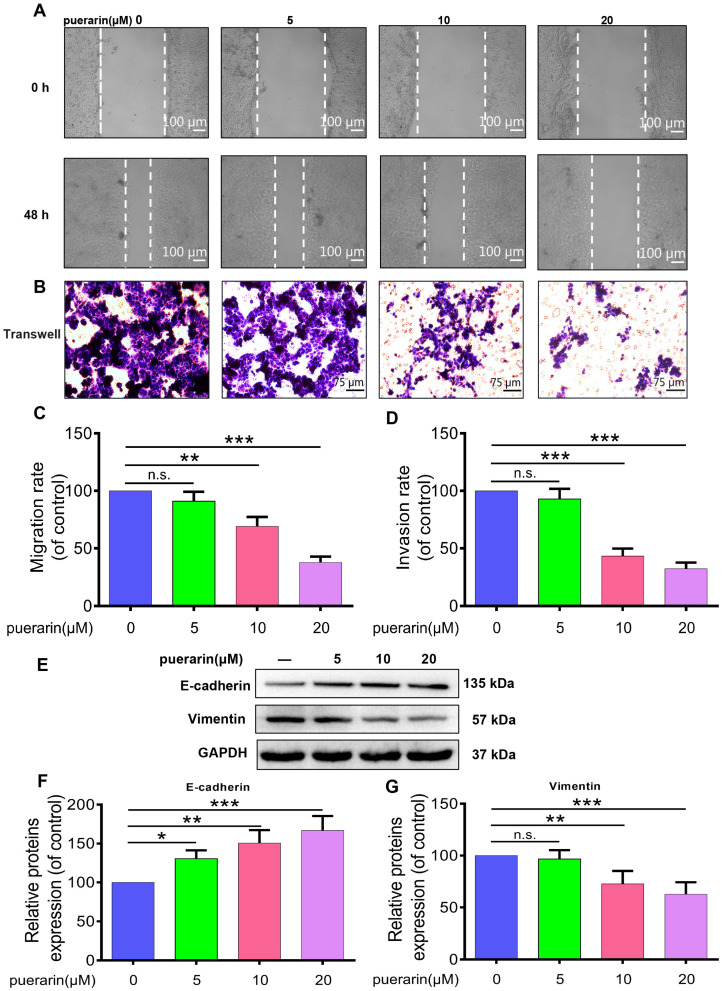
Effects of Puerarin on Caco-2 cell migration, invasion, and EMT markers. (**A**) Scratch assay showing Puerarin (0, 5, 10, 20 μM) effects on Caco-2 cell migration at 0 and 48 h, 50×; scale bar, 100 μm (*n* = 3). (**B**) Transwell assay demonstrating the inhibitory effect of Puerarin on Caco-2 cell invasion, 100×; scale bar, 75 μm (*n* = 3). (**C**) Quantification of migration rate. (**D**) Quantification of invasion rate. (**E**) Western blot analysis showing E-cadherin and Vimentin expression in Caco-2 cells. (**F**) Quantification of E-cadherin expression. (**G**) Quantification of Vimentin expression. Data are presented as mean ± SD. Statistical significance: n.s., not significant; * *p* < 0.05; ** *p* < 0.01; *** *p* < 0.001.

To assess the impact of puerarin on Caco-2 cell migration and invasion, wound healing and Transwell assays were performed (Figure 2A,B). The findings indicated that puerarin effectively inhibited cell migration and invasion, with stronger suppressive effects at higher concentrations (Figure 2A–D). Further investigation of EMT markers revealed that puerarin treatment notably increased E-cadherin expression while significantly reducing Vimentin levels (Figure 2E–G), demonstrating an inhibition of the EMT process. This mechanism attenuates the ability of Caco-2 cells to migrate and invade, highlighting that by blocking EMT, puerarin might serve a crucial role in colon cancer therapy by preventing metastasis. Collectively, these results suggest that puerarin’s modulation of EMT-related pathways contributes to its anti-metastatic potential, offering insights into its therapeutic utility in colon cancer management.

### 2.3. Effects of Puerarin on Apoptosis in Caco-2 Cells

**Figure 3:**
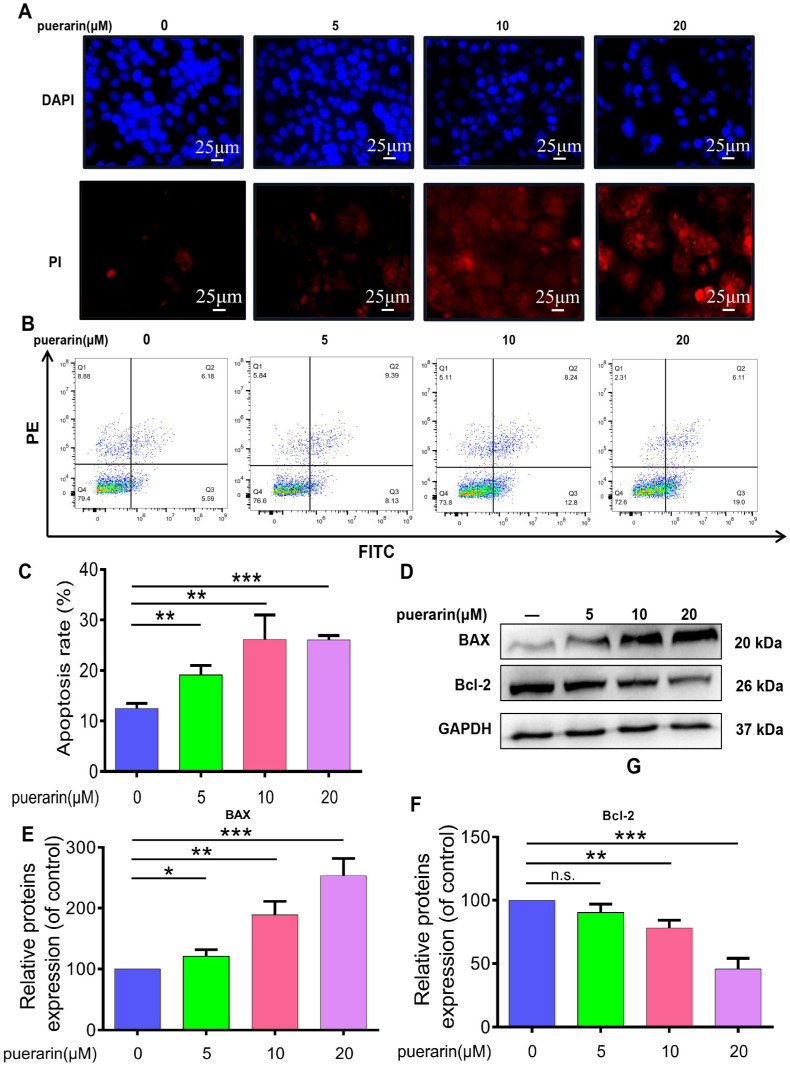
Effects of Puerarin on Caco-2 cell apoptosis. (**A**) DAPI and PI staining showing nuclear condensation and red fluorescence in Caco-2 cells treated with Puerarin (0, 5, 10, 20 μM), 400×; scale bar, 25 μm (*n* = 3). (**B**) Flow cytometry analysis demonstrating a dose-dependent increase in apoptosis rate of Caco-2 cells treated with Puerarin. (**C**) Quantification of apoptosis rate (*n* = 3). (**D**) Western blot analysis showing BAX and Bcl-2 expression. (**E**) Quantification of BAX expression (*n* = 3). (**F**) Quantification of Bcl-2 expression (*n* = 3). Statistical significance: n.s., not significant; * *p* < 0.05, ** *p* < 0.01, *** *p* < 0.001.

Cell morphology was evaluated using DAPI and PI staining (Figure 3A). DAPI staining revealed nuclear condensation and marginalization in Caco-2 cells treated with puerarin, while PI staining showed stronger red fluorescence, indicating membrane damage and apoptosis. Flow cytometry further confirmed a positive correlation between puerarin concentration and the apoptosis rate, with significantly higher apoptosis observed at higher concentrations (Figure 3B,C). The results demonstrated that puerarin treatment markedly increased BAX expression while decreasing Bcl-2 levels, emphasizing its pro-apoptotic effects by promoting apoptotic pathways and inhibiting survival pathways (Figure 3D–F). These findings suggest that puerarin, by modulating BAX and Bcl-2 expression, may inhibit Caco-2 cell proliferation and promote tumor cell apoptosis.

### 2.4. Network Pharmacology Investigation of the Anticancer Mechanism of Puerarin in Colon Cancer

**Figure 4:**
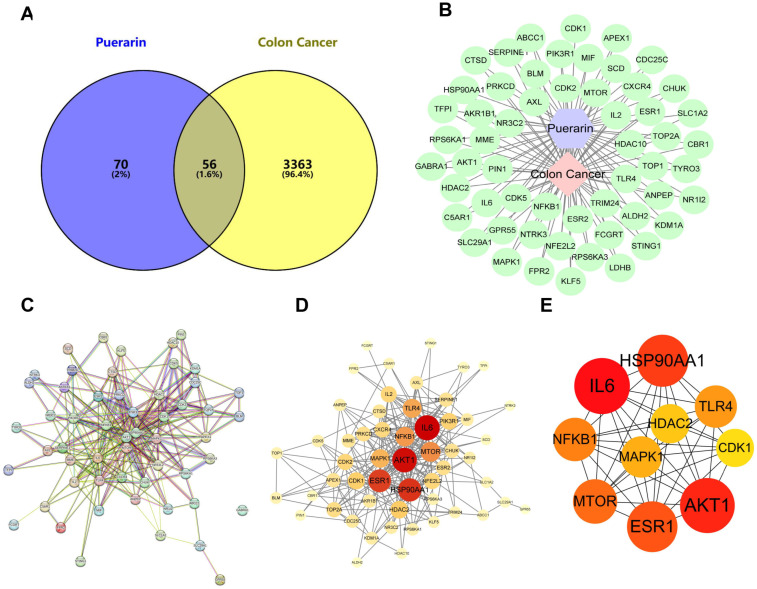

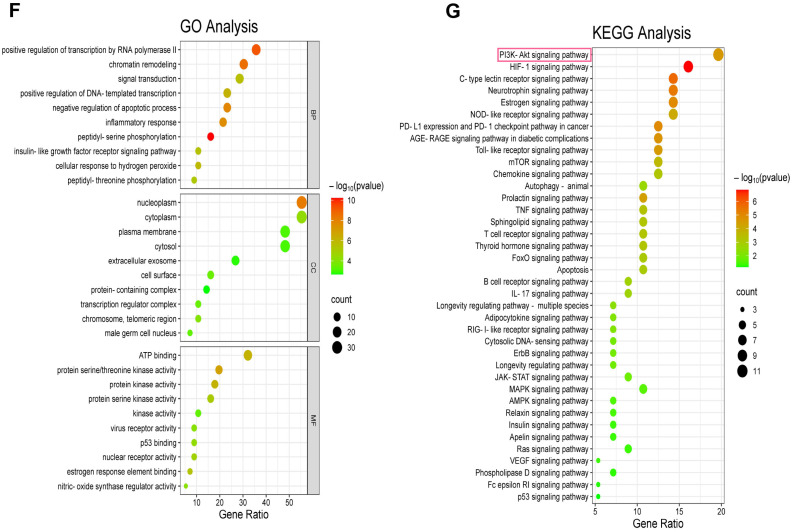
Potential mechanism of Puerarin in colon cancer based on network pharmacology. (**A**) Venn diagram showing the 56 common targets between Puerarin and colon cancer. (**B**) Protein–protein interaction (PPI) network analysis identifying key targets such as *AKT1*, *IL6*, and *HSP90AA1*. (**C**,**D**) Topological analysis of the PPI network showing the central role of these core targets in Puerarin’s therapeutic effects on colon cancer. (**E**) PPI analysis highlighting targets such as *HSP90AA1*, *IL6*, *NFKB1*, *AKT1*, and *MTOR* involved in multiple cancer-related pathways. (**F**,**G**) GO and KEGG enrichment analyses suggesting Puerarin’s regulatory effects on transcription, DNA repair, cell cycle, and signaling pathways such as PI3K-AKT, HIF-1, and mTOR.

To elucidate the underlying anticancer mechanisms of puerarin, a comprehensive network pharmacology analysis was performed, encompassing target prediction, protein–protein interaction (PPI) network construction, and functional enrichment analysis. A Venn diagram (Figure 4A) revealed 56 overlapping targets between puerarin and colon cancer-related genes. The PPI network, visualized through the STRING database (Figure 4B), identified key targets such as *AKT1*, *IL6*, and *HSP90AA1*, which are implicated in colon cancer development. Topological analysis demonstrated that these core targets possess high connectivity and centrality, indicating their potential role in mediating puerarin’s therapeutic effects in colon cancer (Figure 4C–E).

### 2.5. Identification of Colon Cancer Targets

**Figure 5:**
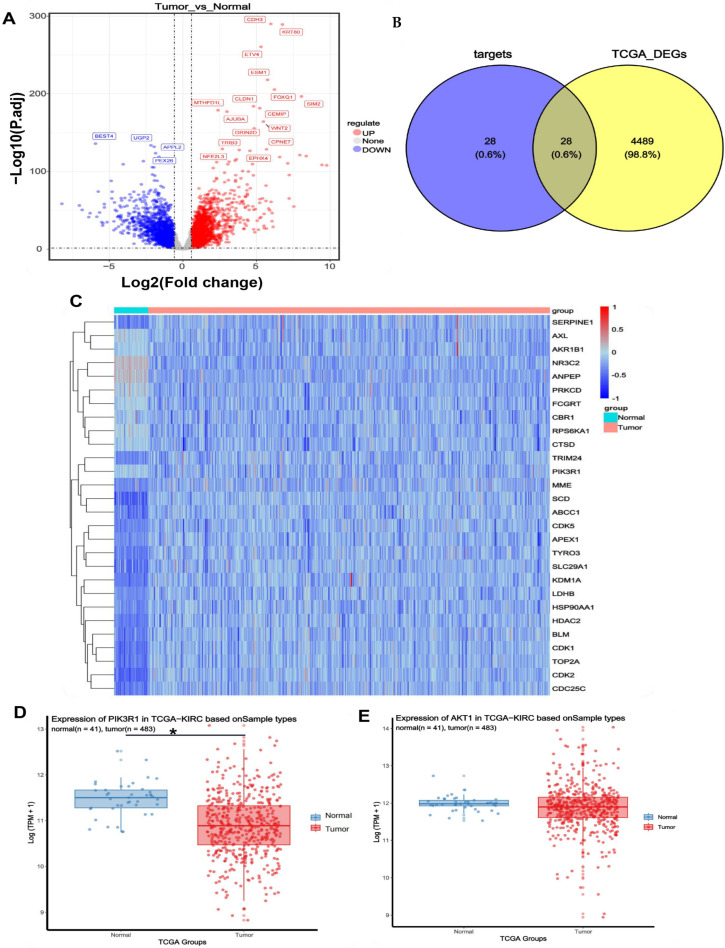
Identification of Colon Cancer Targets. (**A**) Volcano plot showing differentially expressed genes (DEGs) between normal and tumor tissues; (**B**) Venn diagram illustrating the intersection of Puerarin target genes and DEGs; (**C**) Heatmap displaying the expression profiles of 28 intersecting genes in normal and tumor tissues; (**D**,**E**) Expression analysis of key genes *PIK3R1* and *AKT1* in tumor and normal tissues, demonstrating significant downregulation of *PIK3R1* in tumor tissues, while *AKT1* expression showed no significant difference. Statistical significance: * *p* < 0.05.

The acquisition of colon cancer targets was conducted through transcriptomic analysis of the TCGA-COAD (Colon Adenocarcinoma) dataset. RNA-seq data from 41 normal and 483 tumor samples were obtained from The Cancer Genome Atlas (TCGA) database (https://www.cancer.gov/ccg/research/genome-sequencing/tcga) (accessed on 22 February 2025). Differential gene expression analysis was performed using the TCGAbiolinks (version 2.23.0) package to obtain RNAseq count data, with TPM data utilized for visualization. DESeq2 (version 1.44.0) was used to identify differentially expressed genes (DEGs) with selection criteria of padj ≤ 0.05 and |logFC| ≥ 0.58 (|FC| > 1.5). A total of 4517 significant DEGs were identified, including 2583 upregulated and 1934 downregulated genes (Figure 5A).

To determine the potential target genes of puerarin, a Venn diagram was generated using the ggvenn (version 0.1.10) package, showing 28 intersecting target genes (Figure 5B). Heatmap visualization (pheatmap version 1.0.12) of these intersecting genes revealed distinct expression patterns between normal and tumor tissues. Specifically, the *NR3C2*, *ANPEP*, and *FCGRT* genes were significantly upregulated in tumor tissues, while *PIK3R1*, *MME*, *SCD*, and *SERPINE1* genes were notably downregulated (Figure 5C). Analysis of key genes in the PI3K/AKT signaling pathway (*PIK3R1*, *AKT1*) using the ggpubr (version 0.6.0) package indicated that *PIK3R1* was significantly downregulated in tumor tissues, whereas *AKT1* showed no significant difference in expression between normal and tumor samples (Figure 5D,E). Previous studies have reported that *PIK3R1* maintains the homeostasis of the PI3K/AKT signaling pathway by binding to the p110 catalytic subunit of *PI3K* and inhibiting its kinase activity. Downregulation or mutation of *PIK3R1* in tumors weakens this inhibition, leading to PI3K/AKT pathway activation and promoting *AKT* phosphorylation and carcinogenesis [18]. Therefore, we hypothesize that the anticancer effects of puerarin may be mediated through its regulatory action on the PI3K/AKT signaling pathway, particularly by modulating the expression and activity of *PIK3R1*. This suggests that targeting the PI3K/AKT pathway using puerarin could be a promising therapeutic strategy for colon cancer treatment.

### 2.6. Effects of Puerarin on the PI3K/AKT Signaling Pathway in Caco-2 Cells

**Figure 6:**
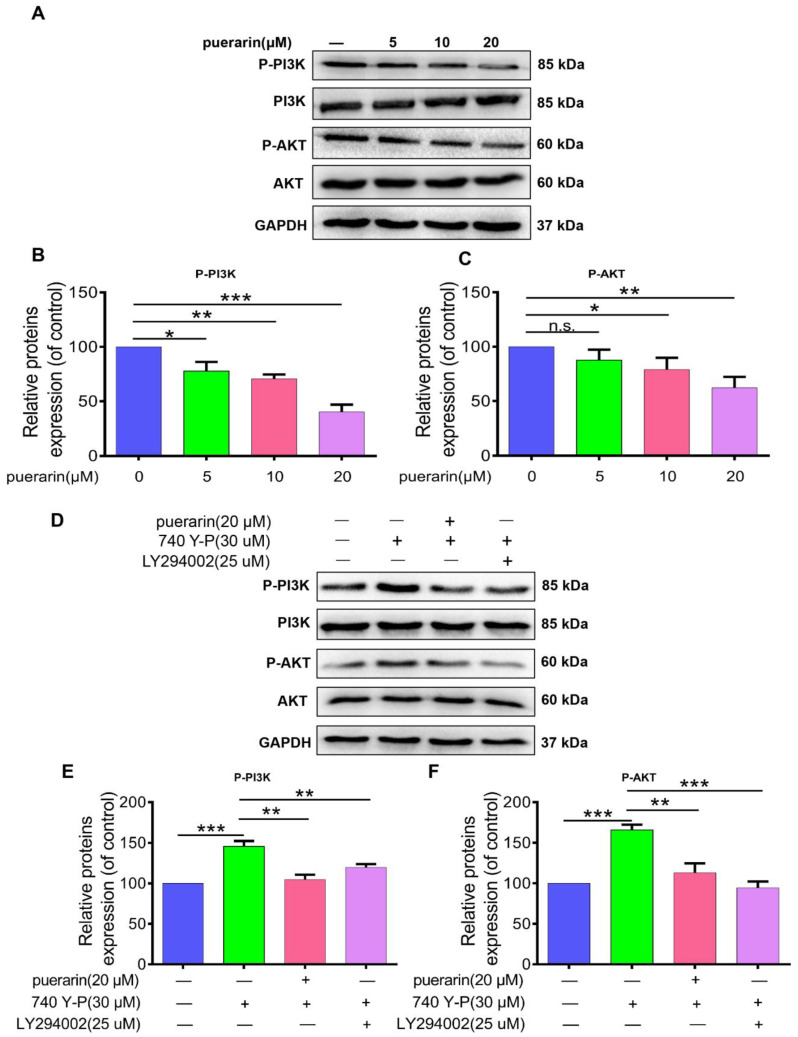
Effects of Puerarin on the PI3K/AKT signaling pathway in Caco-2 cells. (**A**–**C**) Western blot analysis showing reduced phosphorylation levels of PI3K and AKT. Quantification of relative protein expression (*n* = 3). (**D**–**F**) Treatment with PI3K activator 740 Y-P (30 μM) and inhibitor LY294002 (25 μM) showing changes in PI3K and AKT phosphorylation, with representative Western blot images (*n* = 3). Statistical significance: n.s., not significant; * *p* < 0.05, ** *p* < 0.01, *** *p* < 0.001.

To evaluate the impact of puerarin on the PI3K/AKT signaling pathway in Caco-2 cells, Western blotting was performed to measure PI3K and AKT phosphorylation after treatment with puerarin at concentrations of 5, 10, and 20 µM. Puerarin treatment significantly decreased PI3K and AKT phosphorylation in a dose-dependent manner (Figure 6A–C). Further assays using the PI3K activator 740 Y-P (30 µM) and the PI3K inhibitor [19] LY294002 (25 µM) showed that 740 Y-P treatment increased PI3K and AKT phosphorylation compared to controls, while puerarin and LY294002 effectively reversed this effect (Figure 6D–F). These results indicate that puerarin may exert anticancer activity by targeting the PI3K/AKT pathway, leading to reduced cell proliferation and migration in colon cancer cells.

### 2.7. Effects of Puerarin on 740 Y-P-Induced Migration, Invasion, and EMT in Caco-2 Cells

**Figure 7:**
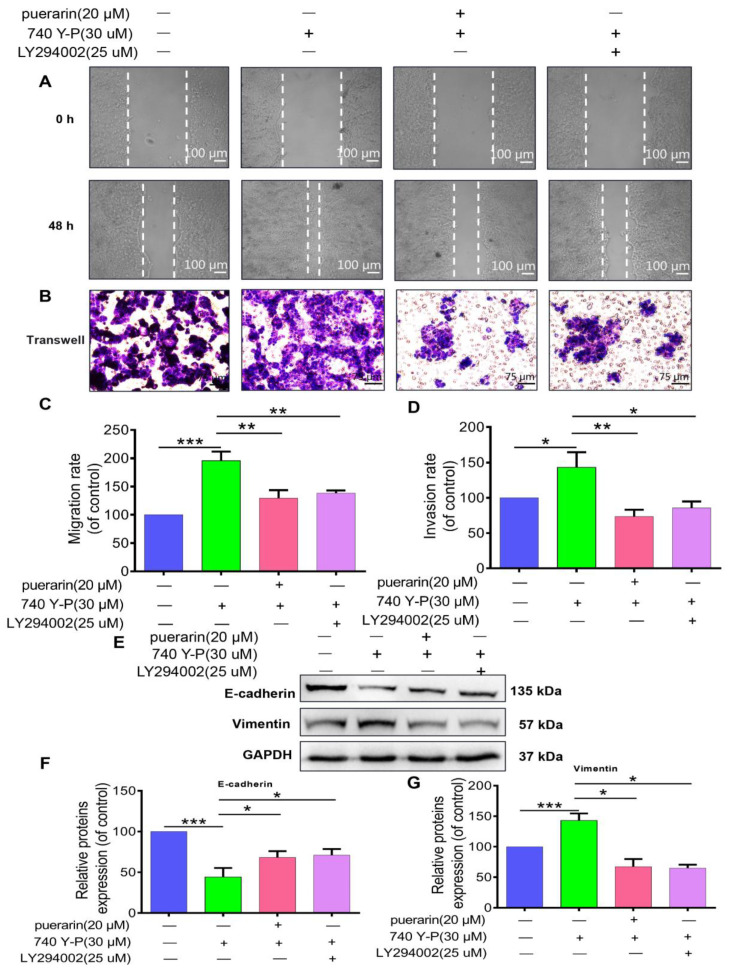
Effects of Puerarin on 740 Y-P-induced Caco-2 cell migration, invasion, and EMT. (**A**) Scratch assay showing 740 Y-P-enhanced Caco-2 cell migration, while Puerarin and LY294002 significantly inhibited migration, 50×; scale bar, 100 μm (*n* = 3). (**B**) Transwell assay demonstrating that 740 Y-P increased cell invasion, while Puerarin and LY294002 treatment significantly inhibited invasion, 100×; scale bar, 75 μm (*n* = 3). (**C**) Quantification of migration rate (*n* = 3). (**D**) Quantification of invasion rate (*n* = 3). (**E**) Western blot analysis showing that 740 Y-P downregulated E-cadherin and upregulated Vimentin, while Puerarin and LY294002 reversed these effects. (**F**) Quantification of E-cadherin expression (*n* = 3). (**G**) Quantification of Vimentin expression (*n* = 3). Statistical significance: * *p* < 0.05, ** *p* < 0.01, *** *p* < 0.001.

To examine how puerarin affects 740 Y-P-induced migration, invasion, and EMT in Caco-2 cells, scratch wound healing and Transwell invasion assays were utilized (Figure 7A). Results demonstrated that 740 Y-P treatment significantly enhanced Caco-2 cell migration and invasion, while puerarin and LY294002 effectively mitigated these effects (Figure 7B,C). Western blot analysis showed that 740 Y-P exposure reduced E-cadherin and increased Vimentin expression in Caco-2 cells. Puerarin and LY294002 treatment reversed these changes, promoting E-cadherin expression and decreasing Vimentin levels (Figure 7D–F). These findings suggest that puerarin, likely through PI3K/AKT pathway inhibition, effectively suppresses 740 Y-P-induced EMT, migration, and invasion in Caco-2 cells, potentially limiting colon cancer cell metastasis.

### 2.8. Effects of Puerarin on 740 Y-P-Induced Apoptosis in Caco-2 Cells

**Figure 8:**
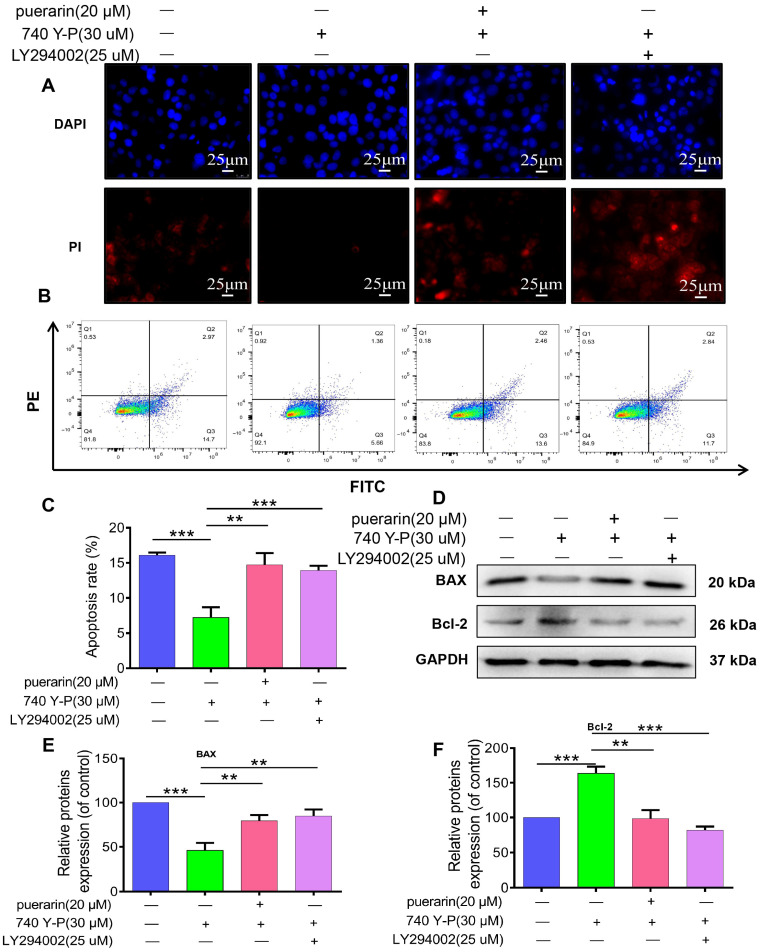
Effects of Puerarin on 740 Y-P-induced Caco-2 cell apoptosis. (**A**) DAPI and PI staining showing that 740 Y-P reduced nuclear condensation and edge aggregation, with weaker red fluorescence. Puerarin and LY294002 restored these changes, 400×; scale bar, 25 μm (*n* = 3). (**B**) Flow cytometry showing that 740 Y-P reduced apoptosis rate, while Puerarin and LY294002 reversed this effect (*n* = 3). (**C**) Quantification of apoptosis rate (*n* = 3). (**D**) Western blot analysis showing that 740 Y-P downregulated BAX and upregulated Bcl-2, while Puerarin and LY294002 treatment upregulated BAX and downregulated Bcl-2, promoting apoptosis. (**E**) Quantification of BAX expression (*n* = 3). (**F**) Quantification of Bcl-2 expression (*n* = 3). Statistical significance: ** *p* < 0.01, *** *p* < 0.001.

Cell morphology was evaluated using DAPI and PI staining (Figure 8A). Caco-2 cells treated with 740 Y-P exhibited reduced nuclear condensation and marginalization, along with decreased red fluorescence, indicating inhibition of apoptosis. In contrast, treatment with puerarin and LY294002 significantly restored apoptotic features, showing enhanced nuclear condensation and membrane damage, suggesting induction of apoptosis (Figure 8A). Flow cytometry analysis further corroborated this observation, revealing that 740 Y-P markedly inhibited apoptosis in Caco-2 cells, whereas puerarin and LY294002 treatments significantly elevated the apoptosis rate, demonstrating their ability to reverse the anti-apoptotic effects of 740 Y-P (Figure 8B). Western blot analysis (Figure 8D,E) showed that 740 Y-P treatment led to reduced BAX expression and increased Bcl-2 levels in Caco-2 cells, suggesting that PI3K activation inhibits apoptosis by altering the BAX/Bcl-2 balance. Conversely, puerarin and LY294002 treatment enhanced apoptosis by upregulating the pro-apoptotic protein BAX and downregulating the anti-apoptotic protein Bcl-2 (Figure 8D,E). These findings indicate that puerarin and LY294002 promote apoptosis by modulating apoptotic proteins and counteracting the PI3K/AKT pathway’s anti-apoptotic effects.

## 3. Discussion

In recent times, increasing research evidence has emphasized the substantial potential of natural bioactive compounds in cancer prevention and treatment [20]. Compared to conventional synthetic drugs, plant- and herb-derived bioactive substances often exhibit higher safety profiles and lower toxicity, offering a promising alternative with reduced risks of adverse effects [21]. Pueraria, a medicinal plant that serves as both food and medicine, is abundant in flavonoids, with puerarin being the most potent and pharmacologically active component. Previous studies have demonstrated that puerarin provides significant therapeutic benefits in cardiovascular and neurological diseases [22,23]. Additionally, current studies indicate that high concentrations of puerarin can effectively inhibit cell proliferation and suppress tumor growth in various cancers, including hepatocellular carcinoma (HCC) [24,25]. In this study, we investigated the effects of puerarin on human colon cancer Caco-2 cells. The experimental findings validated that puerarin treatment significantly diminished Caco-2 cell proliferation in a dose- and time-dependent manner. The IC_50_ values of puerarin displayed variation across different time points, further reinforcing its inhibitory effects on colon cancer cell proliferation. While our study employed sub-cytotoxic doses (5–20 μM) of puerarin to focus on early mechanistic effects and minimize nonspecific cell injury, future experiments at the 48 h IC_50_ concentration (48.47 μM) could provide additional insights into stronger apoptotic responses.

Tumor metastasis is a multifaceted process involving primary tumor detachment, extracellular matrix invasion, penetration into lymphatic and vascular systems, and formation of metastatic lesions in distant organs [26,27,28]. Epithelial–mesenchymal transition (EMT) plays a pivotal role in tumor invasion and metastasis, as it facilitates tumor cell migration, invasion, and dissemination [29,30]. During EMT, tumor cells undergo phenotypic alterations, transitioning from an epithelial to a mesenchymal state. This transformation is marked by the loss of epithelial characteristics, decreased cell adhesion, and increased expression of mesenchymal markers such as N-cadherin and Vimentin, alongside a reduction in E-cadherin expression [30]. In our research, puerarin treatment significantly reduced Caco-2 cell migration and invasion. It also elevated E-cadherin levels while suppressing Vimentin expression, indicating that puerarin may hinder tumor metastasis by modulating the EMT process. Uncontrolled cell proliferation is a hallmark of cancer, and promoting apoptosis in tumor cells to inhibit growth is a crucial strategy in cancer therapy. Many anticancer agents exert their effects by triggering apoptosis, which influences critical cellular behaviors, including migration and invasion [31]. Our study demonstrated that puerarin induced apoptosis in Caco-2 cells, as evidenced by nuclear fragmentation and flow cytometry analysis, reinforcing its potential as an effective therapeutic agent against colon cancer progression.

These results are consistent with prior studies, demonstrating that puerarin alleviates cisplatin-induced apoptosis in hair cells through the mitochondrial pathway and reduces neurological damage in mice via the Bax/Bcl-2, Caspase-3, and Sirt3/SOD2 pathways [32,33]. Bax, a pro-apoptotic protein, and Bcl-2, an anti-apoptotic protein, play vital roles in regulating a cell’s apoptotic susceptibility [34,35,36]. Our findings revealed that puerarin treatment increased Bax expression and reduced Bcl-2 levels, resulting in a higher Bax/Bcl-2 ratio and triggering apoptosis in Caco-2 cells. The PI3K/AKT signaling pathway is crucial for controlling tumor development, including cell proliferation, migration, invasion, and apoptosis [37]. Aberrant regulation of this pathway is closely associated with cancer progression and metastasis [38]. Research suggests that inhibiting this pathway can effectively halt tumor growth and induce apoptosis [39]. Recent evidence highlights that the PI3K/AKT/mTOR signaling axis is instrumental in the onset and progression of colon cancer, with its inhibition offering potential therapeutic benefits [40,41]. In our study, puerarin significantly decreased PI3K and AKT phosphorylation, indicating that it might suppress colon cancer cell proliferation, as well as inhibit migration, invasion, and EMT by disrupting the PI3K/AKT pathway, thereby enhancing apoptosis. Although our network pharmacology analysis identified multiple potential targets (e.g., *AKT1*, *IL6*, *HSP90AA1*), we prioritized the PI3K/AKT pathway for experimental validation due to its central role in colon cancer tumorigenesis and its well-established relevance in related research, over other pathways such as HIF-1 or mTOR. Network pharmacology and KEGG enrichment analyses further identified the PI3K/AKT pathway as a pivotal target of puerarin’s anticancer mechanisms. Additionally, experiments utilizing the PI3K activator (740 Y-P) and inhibitor (LY294002) confirmed that puerarin effectively inhibits cell migration, invasion, and EMT in Caco-2 cells, while promoting apoptosis through the PI3K/AKT signaling inhibition. While these data strongly support that puerarin’s effects are mediated by suppression of the PI3K/AKT signaling pathway, they do not conclusively prove it as the direct or only target. A limitation of this study is the lack of more direct validation methods, such as kinase activity assays or models with constitutively active AKT, which we propose for future work to strengthen mechanistic confirmation. Regarding the TCGA analysis showing downregulation of *PIK3R1* (a regulatory subunit that often acts as a tumor suppressor by inhibiting PI3K activity), we hypothesize that puerarin may upregulate *PIK3R1* expression or function, or alternatively target the catalytic p110 subunits, to modulate the pathway despite this downregulation in tumors. Additionally, as this study is entirely in vitro using a single cell line (Caco-2), which has unique characteristics such as spontaneous differentiation that may limit generalizability, future studies should validate these promising in vitro results in additional colon cancer cell lines (e.g., HCT116, SW480) and in an in vivo xenograft model of colon cancer to evaluate puerarin’s therapeutic potential in a more clinically relevant setting.

## 4. Materials and Methods

### 4.1. Reagents and Chemicals

Puerarin (CAS #3681-99-0) was obtained from Chengdu Dester Biotechnology Co., Ltd. (Chengdu, China). Propidium Iodide (PI, #HY-D0815), 740 Y-P (HY-P0175, 740YPDGFR), and LY294002 (HY-10108) were sourced from MedChemExpress (Shanghai, China). The Annexin V-EGFP/PI apoptosis detection kit (#KTA005) was purchased from Abbkine Scientific Co., Ltd. (Wuhan, China). Matrigel (#356237) was obtained from BD Biosciences (Bedford, MA, USA). Mayer’s hematoxylin (#G1080) and eosin (#G1100) staining solutions were from Solarbio Co., Ltd. (Beijing, China). DAPI (#C0060) was purchased from Solarbio Co., Ltd. (Beijing, China). Anti-E-cadherin antibody (#AF0131, 1:2000) was obtained from Affinity Biosciences (Liyang, China), while Anti-Vimentin antibody (#BS1491, 1:800), Anti-phospho-PI3K antibody (#AP0152, 1:800), Anti-PI3K antibody (#BS3006, 1:1000), and Anti-GAPDH antibody (#AP0063, 1:5000) were purchased from Bio-World (Dublin, OH, USA). Anti-AKT antibody (#9272, 1:900) and anti-phospho-AKT antibody (#4058S, 1:800) were acquired from Cell Signaling Technology (Beverly, MA, USA). Anti-BAX antibody (#A12009, 1:1000) was sourced from ABclonal Biotechnology Co., Ltd. (Wuhan, China), and Anti-Bcl-2 antibody (12789-1-AP, 1:1000) was purchased from Proteintech Group, Inc. (Wuhan, China). Goat anti-mouse (HRP-conjugated, #BS12478, 1:10,000) and goat anti-rabbit (HRP-conjugated, #BS13278, 1:5000) secondary antibodies were acquired from Bio-World (Dublin, OH, USA).

### 4.2. Cell Culture and Proliferation Assay

Caco-2 colon cancer cells were obtained from Shanghai SIBG Biotechnology (Shanghai, China) and cultured in RPMI-1640 medium (Gibco, Grand Island, NY, USA, #C11875500BT) supplemented with 10% fetal bovine serum (FBS) (Wisent, Saint-Jean-Baptiste, QC, Canada, #086-150), 100 U/mL penicillin (Wisent, #450-201-EL), and 100 μg/mL streptomycin (Wisent, #450-201-EL). Cells were maintained at 37 °C in a humidified chamber with 5% CO_2_. For cell proliferation assays, Caco-2 cells (2 × 10^3^ per well) were seeded in 96-well plates and allowed to adhere. Puerarin (0 to 320 μM) was applied for 24, 48, and 72 h. After treatment, 10 μL of Cell Counting Kit-8 (CCK-8, APE×BIO, Houston, TX, USA, #K1018) reagent was added, and absorbance at 450 nm was measured to assess cell viability.

### 4.3. Invasion and Wound Healing Assays

Invasion assays were conducted using Matrigel-coated Transwell chambers (8 μm pores, 24-well format). Caco-2 cells were seeded in the upper chamber with serum-free medium, while the lower chamber contained medium with 10% FBS. After 24 h of incubation at 37 °C with 5% CO_2_, non-invaded cells were removed, and invasive cells were fixed, stained with hematoxylin and eosin, and visualized using a microscope (DMI1; Leica, Wetzlar, Hesse, Germany). ImageJ software (v.1.8.0) was used for quantification. For the wound healing assay, a scratch was introduced into a 95% confluent Caco-2 cell monolayer using a sterile pipette tip. Experimental treatments were applied, and wound closure was observed at 0 and 48 h under an inverted microscope.

### 4.4. Western Blot Analysis

Western blot analysis was conducted as follows: Cells were lysed in RIPA buffer (Beyotime, Shanghai, China, #P0013B) supplemented with protease and phosphatase inhibitors (TransGen Biotech, Beijing, China, #DI101-02). Protein concentration was determined using a BCA assay kit (Beyotime, #P0010). Equal amounts of protein (20–30 μg) were separated by 10–12% SDS-PAGE and transferred to PVDF membranes (Millipore, Burlington, MA, USA, #IPVH00010). Membranes were blocked with 5% non-fat milk in TBST for 1 h at room temperature, then incubated overnight at 4 °C with primary antibodies. After washing with TBST, membranes were incubated with HRP-conjugated secondary antibodies for 1 h at room temperature. Signals were visualized using an ECL detection kit (SparkJade, Jinan, China, #ED0015-C) and quantified with ImageJ software (v1.8.0). GAPDH was used as the loading control.

### 4.5. Immunofluorescence and Apoptosis Assays

Caco-2 cells were treated for 48 h, fixed with 4% paraformaldehyde, permeabilized with 0.2% Triton X-100, incubated with primary antibodies (E-cadherin, Vimentin) and appropriate fluorescent secondary antibodies, and nuclei were counterstained with DAPI; where indicated, propidium iodide (PI) dye (MedChemExpress, #HY-D0815) was used to assess membrane permeability. Images were acquired on a Leica DMI1 microscope and quantified with ImageJ (v1.8.0). For apoptosis analysis, cells were stained with the Annexin V-EGFP/PI apoptosis detection kit (Abbkine, #KTA005) according to the manufacturer’s instructions, incubated for 15 min at 4 °C, and analyzed by flow cytometry to determine apoptotic percentages. 4.6 Puerarin Target Prediction and Colon Cancer-Related Targets.

The SMILES structure of puerarin was obtained from PubChem and analyzed using the Swiss Target Prediction (version 2019), SEA (version 2020), and Super-pred (version 2.0) databases to predict target genes. Colon cancer-related targets were identified through GeneCards (version 5.15), OMIM (version 2023), and DisGeNET (version 7.0) databases, validated using UniProt (version 2023_05), and intersected with puerarin targets using a Venn diagram generated in R (v4.3.1) with the ggvenn package (v0.1.10).

### 4.6. Protein–Protein Interaction (PPI) Network Analysis

Intersection targets were imported into the STRING database (version 12.0; https://en.string-db.org/) (accessed on 25 February 2025) to construct the ‘Drug-Target-Disease’ PPI network with a minimum interaction score of 0.4. Network visualization and topological analysis, including node degree, closeness, and other metrics, were performed using Cytoscape software (v3.9.1; https://cytoscape.org/) (accessed on 25 February 2025) and the CytoHubba plugin (v0.1).

### 4.7. GO Enrichment and KEGG Pathway Analysis

The “Drug-Disease” intersection targets were uploaded to the DAVID database https://davidbioinformatics.nih.gov/ (accessed on 25 February 2025) for Gene Ontology (GO) enrichment analysis, focusing on biological processes (BP), cellular components (CC), and molecular functions (MF). Additionally, KEGG pathway analysis was performed to investigate the mechanisms through which puerarin exerts its effects in colon cancer treatment.

### 4.8. Statistical Analysis

The results are presented as the mean ± standard deviation (SD) derived from at least three independent experiments. Statistical differences between two groups were analyzed using a two-tailed Student’s *t*-test, while comparisons among multiple groups were performed using one-way analysis of variance (ANOVA). Statistical significance was set at * *p* < 0.05, ** *p* < 0.01, *** *p* < 0.001, and “n.s.” indicated no significant difference.

## 5. Conclusions

This study comprehensively examined the anticancer effects of puerarin on Caco-2 cells and uncovered its underlying mechanisms. Our results demonstrate that puerarin significantly inhibits cell proliferation, migration, and invasion, while effectively inducing apoptosis to suppress tumor growth. The anticancer mechanisms of puerarin are primarily associated with the modulation of the PI3K/AKT signaling pathway, where puerarin treatment significantly reduces PI3K and AKT phosphorylation, inhibits EMT, and promotes apoptosis. Additionally, puerarin plays a pivotal role in regulating the Bax/Bcl-2 balance, thereby enhancing apoptosis. In conclusion, puerarin, as a natural bioactive compound, exhibits substantial therapeutic potential in colon cancer treatment, targeting tumor growth and metastasis through multiple molecular pathways. Future research should emphasize evaluating the clinical applicability of puerarin and exploring its synergistic effects when combined with other anticancer therapies.

## Data Availability

Data presented in this study is contained within the article. Further inquiries can be directed to the corresponding author.

## Abbreviations

The following abbreviations are used in this manuscript:

PI3K	Phosphatidylinositol 3-Kinase
AKT	Protein Kinase B
EMT	Epithelial–Mesenchymal Transition
CCK-8	Cell Counting Kit-8
PI	Propidium Iodide
EGFP	Enhanced Green Fluorescent Protein
DAPI	4′,6-Diamidino-2-Phenylindole
PPI	Protein–Protein Interaction
GO	Gene Ontology
KEGG	Kyoto Encyclopedia of Genes and Genomes
TCGA	The Cancer Genome Atlas
COAD	Colon Adenocarcinoma
TPM	Transcripts Per Million
DEGs	Differentially Expressed Genes
SD	Standard Deviation
ANOVA	Analysis of Variance
BAX	Bcl-2-associated X protein
Bcl-2	B-cell lymphoma-2
mTOR	Mammalian Target of Rapamycin
HIF-1	Hypoxia-Inducible Factor 1
FBS	Fetal Bovine Serum
HRP	Horseradish Peroxidase
RPMI	Roswell Park Memorial Institute medium
SMILES	Simplified Molecular Input Line Entry System
IC_50_	Half Maximal Inhibitory Concentration
